# 3D Global Path Planning Optimization for Cellular-Connected UAVs under Link Reliability Constraint

**DOI:** 10.3390/s22228957

**Published:** 2022-11-19

**Authors:** Mehran Behjati, Rosdiadee Nordin, Muhammad Aidiel Zulkifley, Nor Fadzilah Abdullah

**Affiliations:** Department of Electrical, Electronics and Systems Engineering, Faculty of Engineering and Built Environment, Universiti Kebangsaan Malaysia, Bangi 43600, Malaysia

**Keywords:** UAV, drone, path planning, trajectory, cellular connected, cellular network, 4G, link reliability, intelligent optimization, PSO, GA

## Abstract

This paper proposes an effective global path planning technique for cellular-connected UAVs to enhance the reliability of unmanned aerial vehicles’ (UAVs) flights operating beyond the visual line of sight (BVLOS). Cellular networks are considered one of the leading enabler technologies to provide a ubiquitous and reliable communication link for UAVs. First, this paper investigates a reliable aerial zone based on an extensive aerial drive test in a 4G network within a suburban environment. Then, the path planning problem for the cellular-connected UAVs is formulated under communication link reliability and power consumption constraints. To provide a realistic optimization solution, all constraints of the optimization problem are defined based on real-world scenarios; in addition, the presence of static obstacles and no-fly zones is considered in the path planning problem. Two powerful intelligent optimization algorithms, the genetic algorithm (GA) and the particle swarm optimization (PSO) algorithm, are used to solve the defined optimization problem. Moreover, a combination of both algorithms, referred to as PSO-GA, is used to overcome the inherent shortcomings of the algorithms. The performances of the algorithms are compared under different scenarios in simulation environments. According to the statistical analysis of the aerial drive test, existing 4G base stations are able to provide reliable aerial coverage up to a radius of 500 m and a height of 85 m. The statistical analysis of the optimization results shows that PSO-GA is a more stable and effective algorithm to rapidly converge to a feasible solution for UAV path planning problems, with a far faster execution time compared with PSO and GA, about two times. To validate the performance of the proposed solution, the simulation results are compared with the real-world aerial drive test results. The results comparison proves the effectiveness of the proposed path planning method in suburban environments with 4G coverage. The proposed method can be extended by identifying the aerial link reliability of 5G networks to solve the UAV global path planning problem in the current 5G deployment.

## 1. Introduction

### 1.1. UAVs, BVLOS, and Cellular-Connected UAVs

Unmanned aerial vehicles (UAVs), also known as drones, are one of the fastest-emerging technologies. Recently, low-altitude UAVs have received tremendous attention for civil applications, such as surveillance, transportation, environmental monitoring, industrial monitoring, agriculture services, disaster rescue, and goods and medical delivery [1,2,3,4,5,6,7]. UAVs are seen as significant enablers of numerous applications in 5G and 6G networks due to their distinctive features, such as 3D mobility, versatility, and the potential for line-of-sight (LoS) communications [8].

Currently, in most parts of the world, UAV applications are limited to operating within the visual line of sight of pilots [9]. However, for applications such as inspections and package delivery, drones are expected to operate autonomously over long distances, where there is no visual line of sight and the pilot cannot observe the drones during their missions. Hence, the next step in drone technology is to advance drones to fly beyond the visual line of sight (BVLOS).

To ensure a safe flight, drones need stable and reliable wireless connectivity for payload and control and command (CC) communications [10]. One of the limitations of conventional UAV communication systems is their limited communication range. Mainly drone communication can be held through four technologies: direct RF, cellular, satellite, and dedicated links [11]. Among the existing wireless technologies, the cellular network has recently been considered one of the main enablers for ubiquitous UAV communication [11] due to its distinctive features, such as reliability, data rate, latency, and energy efficiency [12].

However, existing cellular networks are designed and optimized for terrestrial users, providing ubiquitous, low-latency, high-speed, and reliable connectivity [13,14]. Providing connectivity to nonterrestrial users, such as flying drones, is challenging. On the one hand, with increasing height above the ground, the radio environment changes, and some problems arise, such as mobility management [15,16] and severe interference between nonterrestrial and terrestrial UEs [17,18]. On the other hand, the transmitting antennas are tilted down, resulting in a severe reduction in the antenna gain at higher heights, which leads to lower link reliability and data rate [15]. Hence, addressing the aforementioned issues and providing robust, reliable, and limitless wireless connectivity for UAVs have become the ongoing topics in beyond 5G and 6G communication [8,19]. Therefore, this paper focuses on identifying the aerial coverage reliability of existing terrestrial 4G networks and optimizing the UAV path planning problem accordingly.

### 1.2. UAV Path Planning

Another crucial issue that needs to be considered in the UAV BVLOS operations is the path planning problem. Flight safety is the most important criterion that needs to be met in a BVLOS operation. The issue of safety is mainly associated with the capability of UAVs to detect and avoid obstacles and communicate payload and CC signals with the ground control station and/or unmanned aircraft systems (UAS) traffic management (UTM) systems. In this regard, appropriate path planning and reliable wireless connectivity play vital roles in enhancing the safety of a UAV along its BVLOS mission.

Path planning involves finding an optimal path between two points by making a tradeoff between several criteria/objects, such as traveling distance, traveling time, power consumption, and safety aspects, such as obstacle avoidance. As such, UAV path planning is considered a complicated optimization problem, in which the object is to find a superior solution (flight route) in a search space (environment) under different constraints [20].

Generally, path planning methods that have been proposed in the literature can be categorized into three groups, as follows:

- Global planning: Global planning intends to find a reference path before performing a mission based on the preexisting knowledge of the environment that can be provided by either users or sensors. The optimal path is considered a set of waypoints that need to be followed by the drone during its mission.
- Local planning: Local methods are used in cases where the considered environment is fully/partially unknown. Therefore, the drone needs to be equipped with onboard sensors and advanced control methods for perceiving the environment, collision avoidance, and real-time path planning. Examples of neural network/machine learning-based techniques for path planning in dynamic and unknown environments can be found in [21,22,23,24].
- Hybrid approach: The hybrid strategy combines the benefits of the two methods mentioned above. In this approach, the drone follows the reference path, and if needed, it will make some amends on the path based on the received data from the environment and the current situation and position of the drone.

The most important factors that should be considered in choosing path planning approaches are their reliability and computational complexity. Global path planning needs to be conducted prior to a flight; hence, algorithms can be run in an external processing unit. The dependability of such methods in dynamic and unpredictable situations is a major problem, even though computing complexity is not crucial for global approaches. Meanwhile, although local methods are superior in path planning in unknown and dynamic environments, they are slow in environmental mapping and decision making and computationally expensive, especially for onboard computation [25]. In addition, by relying only on local approaches, path planning will suffer from being trapped in local minima. In this case, a global path can help to get out of local minima and converge to global optima. Therefore, a combination of global and local techniques can help enhance the reliability of path planning and reduce the computational complexity in autonomous UAV operations.

### 1.3. Motivation and Contributions

As discussed above, one of the critical challenges to enhance BVLOS flight safety is the lack of reliable and ubiquitous aerial wireless communication systems. Motivated by the distinctive features of existing cellular networks, partially provided aerial coverage, adaptability of UAVs, and the important role of global path planning approaches to facilitate path planning for autonomous UAV operations, this paper proposes a practical method to find a 3D collision-free global path for UAVs operating BVLOS.

The objectives of the path planning optimization problem are to minimize the flight distance and maximize the cellular link reliability while satisfying real-world constraints, such as power consumption and permitted flight height. The key contributions of this work can be summarized as follows:

- A reliable aerial 4G coverage zone for UAV communications in suburban environments has been proposed. The reliable zone has been identified based on the statistical analysis of 4G key performance indicators (KPIs), such as reference signal received power (RSRP), reference signal received quality (RSRQ), and the number of handovers, which have been extensively measured in a suburban environment.
- A cumulative objective function has been developed for the 3D UAV path planning optimization. The objective function consists of the length of the total path, the length of the reliable path, and a set of penalty functions for cases where the path collides with the obstacles and passes from the forbidden zones. Furthermore, other real-world constraints, such as maximum and minimum permitted flight height and power constraints, have been applied to the optimization problem.
- The performance of the proposed UAV path planning method has been investigated by utilizing different intelligent optimization algorithms, such as particle swarm optimization (PSO) and genetic algorithm (GA), and a combination of both algorithms, PSO-GA. Furthermore, the performances of the considered optimization algorithms for solving 3D path planning problems have been investigated under different scenarios.
- The ability of the proposed strategy to solve real-world 3D path planning problems is validated by comparing the simulation results with the aerial drive test results. The comparison results prove that the proposed strategy can provide a stable and valid global optimal solution for UAV path planning in suburban environments.

The remainder of this paper is organized as follows: A review of recent related works is presented in Section 2. Section 3 describes the proposed UAV path planning strategy and research methodology, including the measurement method, identifying reliable cellular network aerial coverage, as well as defining the path planning problem, objective functions, search space, and constraints. Section 4 presents the measurement results of the aerial drive test in a 4G network and discusses the reliable aerial coverage. Next, the simulation results of the path planning optimization under different scenarios are discussed. Then, the performance of the proposed strategy is validated by comparing the simulation results with the aerial drive test results. Finally, Section 5 concludes the paper.

## 2. Related Works

Generally, two types of algorithms have been used for solving global path planning problems: classical and intelligent algorithms. The former includes the Dijkstra algorithm [26], the A-star algorithm [27], simulated annealing [28], and differential evolution [29]. However, the main shortcomings of such algorithms are their need for large memory and extended run time. Meanwhile, intelligent optimization algorithms, such as evolutionary and swarm intelligence algorithms, have become more popular for solving path planning problems due to their inherent features and design principles, such as flexibility, robustness, high search capability, and accuracy.

Over the past decade, different intelligent optimization algorithms have been utilized to solve path planning problems in robotics, such as ant colony algorithm [30], firefly algorithm [31], artificial bee colony [32], GA [33], PSO [34], and whale optimization algorithm [35]. In addition, heuristics intelligent optimization algorithms have also been widely used to solve local path planning optimization problems, such as graph-based algorithms [36], heuristic search algorithms [37], field-based algorithms [38], and intelligent optimization algorithms [39]. Most of the algorithms utilized to solve UAV path planning problems have originally adapted the existing algorithms for robot path planning. Articles such as [40,41] thoroughly surveyed the most commonly used path planning algorithms.

The authors in [42] proposed an improved crossover operation for solving 2D path planning problems for autonomous mobile robots using GA in a static environment. In [43], the wolf pack algorithm was utilized to solve the multiobjective 3D path planning problem for drones. The authors improved the algorithm performance by applying the mutation and crossover operations of GA to the wolf pack algorithm. Ref. [44] proposes a UAV path planning algorithm based on the random tree planning technique while accommodating real-time traffic and geofencing constraints. The proposed algorithm can terminate tree expansion based on the defined criteria, resulting in lower computational complexity and making the algorithm suitable for onboard execution.

The authors in [45] formulated a UAV trajectory problem to minimize the flight time while ensuring the outage performance during the mission. To reduce the problem complexity, a low-complexity method was proposed to find the shortest path in an undirected weighted graph with enlarged cellular coverage. Ref. [46] considers the effect of 3D antenna radiation patterns and backhaul constraints on the path planning of UAV-assisted wireless networks. It was considered that the UAV acts as a relay and the object to optimize the trajectory for improving the wireless coverage for terrestrial uses.

The authors in [47] utilized the PSO algorithm to maximize the throughput of a UAV-assisted wireless network by adjusting the flight height and transmission power of the UAV. The simulation results showed that the PSO can rapidly converge to an optimal solution for the considered problem. In [5], an enhanced version of PSO (EPSO) was utilized for UAV path planning in a large-scale remote environment. The results showed the effectiveness of EPSO in solving the UAV path planning for aerial-based data collection in a wireless sensor network. Table 1 presents a summary of the reviewed studies with a description of the application, optimization approach, key findings, and limitations.

Based on the results of the reviewed literature, it can be seen that more research is still needed on 3D global path planning to enhance the safety of UAVs, especially for BVLOS operations. In addition, despite the popularity and capability of intelligent optimization algorithms in solving path planning problems, the algorithms still need to overcome some shortcomings, such as being trapped in local optima and early convergence, especially when the dimension and complexity of problems increase. Furthermore, there are only a few articles on the optimization of UAV path planning based on cellular communication objectives/constraints. Finally, there is a gap in proposing an optimization method for the 3D global path planning in the presence of obstacles and the realistic constraints of the terrestrial cellular networks in providing reliable aerial coverage.

On the other hand, the intelligence optimization algorithms, such as particle swarm optimization (PSO) and genetic algorithm (GA), have been widely utilized to solve path planning problems. Although in 2D cases their performance is stable and converges to the global optima, when increasing the problem complexity, the algorithms suffer from their inherent shortcomings, such as being trapped in local minima and premature convergence. To address these issues, a combination of PSO and GA (here referred to as PSO-GA) is utilized in this study. The PSO-GA algorithm advantages the information flow of PSO as well as the crossover and mutation operations of GA.

## 3. Methodology

**Figure 1:**
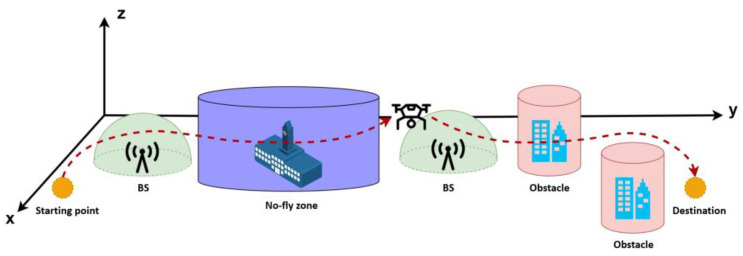
Schematic diagram of the considered path planning problem.

**Figure 2:**
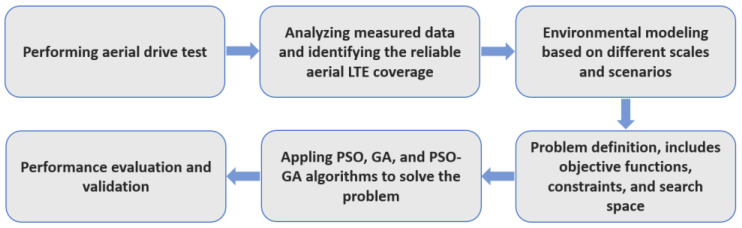
General flowchart of the methodology.

In this study, it is considered that a UAV is moving in a 3D workspace, ${ℛ}^{3}$, where a set of BSs, obstacles, and no-fly zones are distributed over the workspace. Given the initial and destination and the locations of BSs, obstacles, and no-fly zones, the problem consists of finding an optimal path in the workspace that avoids collision with the objects, avoids passing from no-fly zones, minimizes the total traveling distance, and maximizes communication link reliability. Figure 1 depicts a schematic diagram of the considered problem. Figure 2 depicts the general flowchart of the methodology, and the following subsections describe each step in detail.

### 3.1. Field Measurement

To study the reliability of cellular communication aerial links, we conducted a comprehensive measurement campaign in a 4G network within the National University of Malaysia (UKM), Bangi campus, Malaysia. The campus environment can be considered a suburban metropolitan area with a geographic terrain of undulating hills. The dataset is publicly accessible on [48]. To measure the required cellular key performance indicators (KPIs), the G-NetTrack Pro application [49] was installed on a Huawei STK-L22 smartphone, and the smartphone was mounted on the developed drone in [50]. During the measurement, the drone flew at different altitudes and routes, the smartphone was served by the 4G mobile network, and G-NetTrack Pro measured the 4G-related parameters, such as reference signal received power (*RSRP*) and reference signal received quality (*RSRQ*). For details on the development of the drone and the method of conducting the aerial drive test, readers are referred to [50,51], respectively.

*RSRP* is a key measurement parameter indicating the average received signal power of a single resource element in an LTE Resource Block (RB) and can be calculated as [52]

(1) $$RSRP\left[W\right]=\frac{1}{N}\overset{N}{\underset{n=1}{\sum }}P_{n\;},$$

where N is the number of received reference signals and $P_{n\;}$ is the received power of the $n^{th}$ reference signal. However, *RSRP* alone does not fully reflect the quality of the received signal because it also picks up the energy of interfering signals in the corresponding frequency range.

*RSRQ* is another key measurement parameter that indicates the received signal quality level in the 4G network and the effect of interference from adjacent BSs. *RSRQ* can be calculated as [52]

(2) $$RSRQ=N\times \frac{RSRP\left[W\right]}{RSSI\left[W\right]},$$

where Reference Signal Strength Indicator (*RSSI*) is the power measured over the entire bandwidth of occupied RBs, including intracell power, interference, and noise. Note that *RSRQ* is dimensionless and usually written in dB.

**Figure 3:**
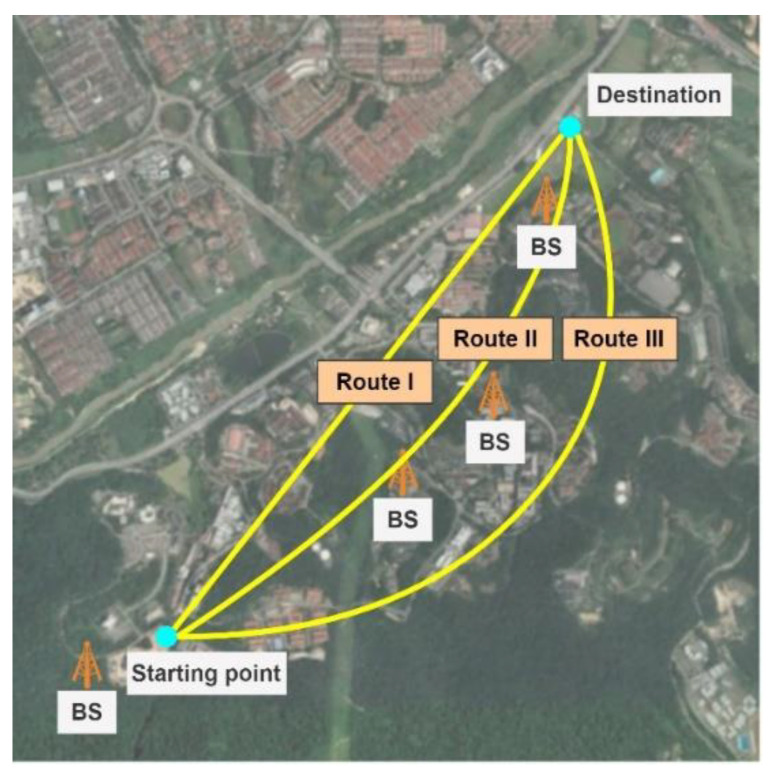
Illustration of the conducted aerial drive test on the UKM campus in three different routes.

Figure 3 depicts the different examined routes and the location of the serving BSs inside the campus. The drive tests were conducted on three different routes and at four different elevations (65, 85, 105, and 125 m). The measured data are statistically analyzed in this step to identify reliable aerial communications coverage for cellular-connected drones. The output of this step is used as the input to define the objective function of the path planning problem in Section 3.2.2.

### 3.2. Problem Definition and Formulation

#### 3.2.1. Environmental Modeling

In this study, three types of environments were modeled. The models were created based on the environment scale and the presence of obstacles and no-fly zones. All three scenarios were generated based on real-world environments in a suburban environment in Bangi, Malaysia. Table 2 lists the considered parameters in modeling the environments. The considered scenarios are expanded from small-scale scenario (Scenario I) to large-scale scenario (Scenario II) and, finally, to Scenario III, which represents a large-scale scenario with more realistic assumptions related to BVLOS UAV operations. The terrain is considered flat to reduce the computation complexity and maintain this paper’s main objective. However, the terrain also can be simulated by numerical methods, where the train profile can be presented in the form of a matrix, in which matrix elements contain the terrain elevation in their respective coordinates.

**Figure 4:**
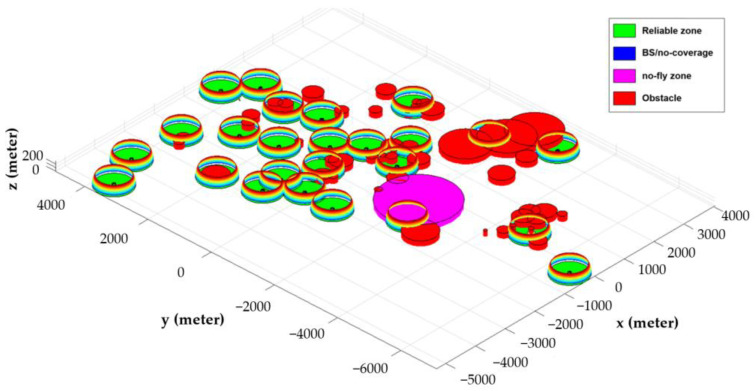
3D representation of the simulation model for Scenario III in MATLAB environment.

Figure 4 depicts a simulated model for Scenario III, where the partial hemispheric represents the reliable cellular coverage zone, the red cylinder represents the obstacles (such as high-rise buildings, hills, water reservoirs, etc.), and the violet cylinder represents the no-fly zone.

#### 3.2.2. Objective Function

The path planning problem in this study is considered a dynamic multiobjective problem with a deterministic objective function and a vector output, ${ℛ}^{3}$. The output is a set of 3D waypoints, which is a combination of three nonlinear functions along three coordinate axes. Since the goal is to minimize the objective function, hereafter, we use the term cost function instead of objective function. The cost function is defined as

(3) $$F=L_{total}-\alpha \times L_{reliable}+\beta \times P_{nc}+\gamma \times P_{ob}+\delta \times P_{nf},$$

where $L_{total}$ is the total length of the computed path between the starting and end points. $L_{reliable}$ is a function of the path length with a reliable communication link. $P_{ob}$, $P_{nc}$, and $P_{nf}$ are the penalty terms, while the path collides with the obstacles and passes from the no-coverage and no-fly zones, respectively. The penalty terms are defined based on the problem constraints, which will be discussed later. α, β, γ, and δ are the determination of weighted coefficients.

The solution for the path planning problem is a function of x(t), y(t), and z(t), which correspond to the drone coordinates in space. Generally, these functions are continuous in time; therefore, infinite variables need to be considered to solve the problem. One common approach to reducing the problem complexity is using the spline interpolation technique, in which the problem can be solved with a limited number of variables, and the spline function can produce a smooth path. Therefore, the output of the path planning problem is a function of x, y, and z, which is a vector of size k, that contains computed 3D waypoints in space, where k is equal to the number of query points in the spline function.

$L_{total}$ can be computed as

(4) $$L_{total}=\sum^{\;}\sqrt{diff{\left(x\right)}^{2}+diff{\left(y\right)}^{2}+diff{\left(z\right)}^{2}},$$

where diff is the difference function that calculates differences between adjacent elements of a vector, as [x(2)−x(1), x(3)−x(2),…,x(k)−x(k−1)].

One approach to modeling the wireless link reliability objective function is based on the wireless channel’s characteristics. Such a function would be a dynamic and stochastic function, with a random distribution; therefore, the output also would be dynamic and random. To optimize this objective function, both mean and variance need to be optimized simultaneously. Since the output is not deterministic, the objective needs to be optimized with a probability, which is challenging.

Inspired by reliable zone selection in terrestrial cellular communications and to reduce the complexity of the path planning problem, we identified a reliable aerial coverage zone for cellular-based drone communications by statistically analyzing the measured data described in Section 3.1. Based on the described strategy, $L_{reliable}$ is defined as

(5) $$L_{reliable}=\sum^{\;}r_{mask}\times \sqrt{diff{\left(x\right)}^{2}+diff{\left(y\right)}^{2}+diff{\left(z\right)}^{2}},$$

where $r_{mask}$ is a mask vector of size k, where its elements are either 1 or 0. If a computed point is within the reliable zone, its corresponding element in $r_{mask}$ is 1; otherwise, it is 0. $r_{mask}$ is defined as

(6) $$r_{mask}=r_{i}\cup r_{j}\;\;\;\;\;\forall i,j\in n_{BS}\;and\;i\neq j,$$

where $n_{BS}$ is the number of BSs and r is the reliable zone function, which is defined as

(7) $$r=\max\left(1-\frac{d_{BS}}{r_{rz}},0\right)\times \left(\frac{r_{rz}}{r_{rz}-d_{BS}}\right),$$

where $r_{rz}$ is the radius of the reliable zone, and $d_{BS}$ is the 3D distance between the computed point and a BS, for example, for the $i^{th}$ point, it can be calculated as

(8) $$d_{BS}=\sqrt{{\left(x_{d}^{i}-x_{BS}\right)}^{2}+{\left(y_{d}^{i}-y_{BS}\right)}^{2}+{\left(z_{d}^{i}-z_{BS}\right)}^{2}},$$

where $x_{BS}$, $y_{BS}$, and $z_{BS}$ are the coordinates of the considered BS antenna in space, and $x_{d}^{i}$, $y_{d}^{i}$, and $z_{d}^{i}$ are the coordination of the $i^{th}$ point at space.

The penalty terms for no-coverage zones, obstacles, and no-fly zones are defined as

(9) $$P_{nc}=\underset{n_{BS}}{\sum }\max\left(1-\frac{d_{BS}}{r_{nc}},0\right),$$

where $r_{nc}$ is the radius of the spherical zone around the BS that there is no reliable coverage. It is generally accepted that the shorter the distance to the BS, the higher the probability of serving users with reliable signals. Meanwhile, in a close range to the BSs center, the signal quality is unreliable due to the antenna propagation pattern, especially in the boundaries of the main lobe. Another reason to consider this parameter is to avoid collision with the BS tower,

(10) $$P_{ob}=\underset{n_{ob}}{\sum }\max\left(1-\frac{d_{ob}}{r_{ob}},0\right),$$

where $d_{ob}$ is the distance of the computed point to the center of the obstacle, $r_{ob}$ is the radius of the obstacle, and $n_{ob}$ is the number of obstacles in the problem,

(11) $$P_{nf}=\underset{n_{nf}}{\sum }\max\left(1-\frac{d_{nf}}{r_{nf}},0\right),$$

where $d_{nf}$ is the distance of the computed point to the center of the no-fly zone, $r_{nf}$ is the radius of the no-fly zone, and $n_{nf}$ is the number of no-fly zones in the problem.

#### 3.2.3. Search Space and Constraints

It is well known that constraints enhance the problem complexity. Hence, we either (i) applied constraints as penalty functions into the cost function, such as Equations (9)–(11), or (ii) applied constraints as search space limitations, as described below.

The coordinates of the space are denoted as x, y, and z; hence, the search space can be expressed as

(12) $$\left\{\left(x,y,z\right)\in ℛ|x_{min}\leq x\leq x_{max},y_{min}\leq y\leq y_{max},z_{min}\leq z\leq z_{max}\right\},$$

where, $x_{min},x_{max},y_{min},y_{max},z_{min}$, and $z_{max}$ define the boundary of x, y, and z, respectively. The boundaries of x and y can be defined according to the scale of the considered environment. The constraint of the permitted UAV flight height is applied to the boundaries of z. Hence, $z_{min}$ and $z_{max}$ can be defined based on the UAV flight regulations in each region/country.

Another constraint that needs to be taken into account is the maximum flight distance, $L_{max}$, the maximum distance a drone can travel in a single flight. $L_{max}$ is mainly limited due to physical constraints, such as battery and fuel restrictions. Therefore,

(13) $$L_{total}\leq L_{max},$$

where $L_{max}$ for a battery-powered multirotor can be estimated as

(14) $$L_{max}=V_{avg}\times \frac{C_{battery}\times D_{battery}}{AAD}\;\left(\mathrm{in}\;\mathrm{km}\right),$$

where $V_{avg}$ is the drone’s average speed in km/h, $C_{battery}$ is the battery capacity in Ah, $D_{battery}$ is the battery discharge ratio, and AAD is the drone’s average ampere draw in amps. In this study, $D_{battery}$ is considered 0.8, since it is common practice not to discharge LiPo batteries below 20% mAh during flight. The calculation of AAD depends on factors such as the drone’s payload, the size of motors, and utilized hardware components. The details of AAD of the utilized drones in this study can be found in [50].

### 3.3. Optimization Algorithms

Intelligence optimization algorithms, such as PSO and GA, have been widely applied in path planning optimization problems. In this study, we used PSO, GA, and a combination of PSO and GA due to their advantages, such as strong robustness, simulation evolution, and notable exploration and exploitation capability. The following describe the algorithms and their adjustments in detail.

#### 3.3.1. Particle Swarm Optimization

In the PSO algorithm, first, all particles are scattered randomly in the search space, and every particle calculates the objective function based on its position in the search space. Then, each particle computes its next movement direction based on a combination of information about its current position, the best position it has experienced so far, its current velocity, and information from one or more of the best particles in the swarm. Then, particles move, one step of the algorithm ends, and in case of necessity, the above steps are iterated until the algorithm meets the termination criteria.

To formulate the behavior of particles, assume that there are $\;n_{pop}$ particles in the swarm, where the position and velocity of the $i^{th}$ particle at time  t are denoted as $x^{i}$ and $v^{i}$, respectively, for i∈{}. $x^{i,\;best}\left[t\right]$ is the best position that the $i^{th}$ particle has experienced so far, and $x^{gbest}\left[t\right]$ is the position of the swarm’s best experience. In every iteration, the swarm updates its best position (based on objective value), which is known as global best; also each particle updates its best solution (aka personal best) and computes its next position as follows:

(15) $$v^{i}\left[t+1\right]=wv^{i}\left[t\right]+c_{1}r_{1}\left(x^{i,\;best}\left[t\right]-x^{i}\left[t\right]\right)+c_{2}r_{2}\left(x^{gbest}\left[t\right]-x^{i}\left[t\right]\right),$$

(16) $$x^{i}\left[t+1\right]=x^{i}\left[t\right]+v^{i}\left[t+1\right],$$

where w is the inertia coefficient; $c_{1}$ and $c_{2}$ are cognitive and social acceleration coefficients, respectively; and $r_{1}$ and $r_{2}$ are random numbers with a uniform distribution, $r_{1},r_{2}\sim u\left(0,1\right)$. The adjustment of w, $c_{1}$, and $c_{2}$ plays an important role in the performance of the PSO algorithm, which directly affects the convergence speed of the algorithm to the best cost function.

Small values of w result in rapid convergence and enhance the risk of trapping in a local minimum, and large values of w result in random behavior or particles. In other words, a small value of w helps exploitation, and a large value of w helps exploration. Exploration is the capability of finding new solutions, and exploitation is the capability of developing/improving existing solutions. A pure exploration results in a random search, and a pure exploitation results in a local search. In addition, small values of $c_{1}$ and $c_{2}$ help exploitation, and large values of $c_{1}$ and $c_{2}$ help exploration. The coefficients need to be appropriately set to make a tradeoff between exploration and exploitation. To set the coefficients efficiently, we used the proposed constriction coefficients by Clerk [53] as

(17) $$\begin{array}{c} w=\chi ,\\ c_{1}=\chi \phi_{1},\\ c_{2}=\chi \phi_{2}, \end{array}$$

where $\phi_{1},\phi_{2}>0$ and $\phi \equiv \phi_{1}+\phi_{1}>4$, and

(18) $$\chi =\frac{2}{\phi -2+\sqrt{\phi^{2}-4\phi }}.$$

According to [54], the optimal values for the above parameters are $\phi_{1}=\phi_{2}=2.05$, w=0.7298, and $c_{1}=c_{2}=1.4962$.

To improve the performance of the algorithm, the inertia weight damping ratio, $w_{damp}$, was added into the algorithm as

(19) $$w=w\times w_{damp},\;\mathrm{where}\;w_{damp}<1,$$

in which, first, the algorithm starts with a high exploration rate. By updating w at the end of each iteration, w gradually decreases, which consequently reduces the exploration rates and enhances exploitation ability. In simulations, $w_{damp}$ was set to 0.99.

The velocity limitation was also applied to the algorithm as

(20) $$V_{min}<v^{i}\left[t\right]<V_{max},$$

where

(21) $$V_{max}=-V_{min}=0.1\times \left(x_{max}-x_{min}\right).$$

In the simulation, the number of function evaluations (NFE) was used as the termination criteria, in which the PSO algorithm stops its execution when meeting the criteria. NFE can be defined as

(22) $$NFE\left(t\right)=n_{pop}+n_{pop}\times n_{it}=n_{pop}\left(1+n_{it}\right).$$

Algorithm 1 presents the pseudo-code of the PSO algorithm, and Table 3 lists the general parameters in the simulation scenarios. Since Scenarios II and III environmental scales are the same, the general simulation parameters are considered to be the same.

**Algorithm 1.** Particle swarm optimization.1:**Input:**$n_{pop}$: swarm size; $n_{var}$: no. of variables; $n_{it}$: maximum no. of iterations; w: inertia weight; $c_{1},\;c_{2}$: acceleration coefficients2:**Output:** Best solution3:**for**$i\;=\;1:n_{pop}$**do** (initialize the parameters)4:  Randomly generate *n* initial positions $X^{i}\left(i=1,2,\ldots ,n\right)$ of $n_{pop}$ particles5:  Set *n* initial velocities
$V^{i}\left(i=1,2,\ldots ,N\right)$ of $n_{pop}$ particles to 06:  Calculate the cost value of each particle7:  Set $P_{best}$ and $g_{best}$ in the swarm8:
**end for**
9:**for** i = 1: $n_{it}$
**do**10:  **for**
$i\;=\;1:n_{pop}$
**do**11:    Update $V^{i}$ of the $i^{th}$ particle using Equation (15)12:    Update velocity bounds using Equation (20)13:    Update $X^{i}$ of the $i^{th}$ particle using Equation (16)14:    Apply velocity bounds15:    Calculate the cost value of the new particle $X^{i}$16:    **if**
$X^{i}$ is superior to $P_{best\_i}$17:      Set $X^{i}$ to be $P_{best\_i}$18:      **if**
$X^{i}$ is superior to $g_{best}$19:        Set $X^{i}$ to be $g_{best}$20:      **end if**21:    **end if**22:  **end for**23:  Check the feasibility of the solution by checking Equations (9)–(11) = 024:  Update best cost ever found, $g_{best}$25  Update the inertia weight using Equation (19)26:
**end for**


#### 3.3.2. Genetic Algorithm

GA is the most essential evolutionary algorithm, which has been inspired by the Darwinian evolution theorem concepts involving an initialization method, objective function to evaluate each chromosome, natural selection, crossover, and mutation operators. Conventionally, GA has been used as an effective method to solve the 2D path planning problem and helps to find an optimal global path for many robotic problems.

The GA algorithm initially starts with generating a random set of populations, representing possible solutions for the optimization problem. Each solution needs to be evaluated by an objective function to qualify the generated solutions. In the next step, a selection operation is used to choose the parents that are subjected to reproduction according to their objective function value. Later, the crossover operation is applied to produce new progenies by recombining data from the two parents selected in the previous step. Another genetic operation that is applied is the mutation operation, which is used to enhance the diversity of the population by changing the genetic structure of parents based on a mutation rate. This procedure is repeated until the termination criteria are satisfied.

The parent selection criteria for crossover and mutation are based on the merit of individuals, in which individuals with better cost values have a higher chance of being selected for crossover and mutation. As such, the Boltzmann method is used to calculate the selection probabilities associated with each individual:

(23) $$p_{i}=\frac{e^{-\eta \frac{c_{i}}{c_{max}}}}{\sum_{j=1}^{n_{pop}}e^{-\eta \frac{c_{j}}{c_{max}}}},$$

where $\overset{n_{pop}}{\underset{i=1}{\sum }}p_{i}=1$, $c_{i}$ is the cost value corresponding to the $i^{th}$ individual, and $c_{max}$ is the worst cost value of the population, which is added for normalization purposes. η is the selection pressure. If η=0, the selection probabilities of all individuals are the same, which is equivalent to a random selection, where $p_{i}=\frac{1}{n_{pop}}$ $\forall i\in n_{pop}$. If η→∞, the selection probability of the best individual is one, and the selection probability of the rest of the population is zero. In this study, the roulette wheel selection (RWS) method [55] was used for population selection.

Let us define $p_{c}$ and $p_{m}$ as crossover and mutation rate; then the number of offspring, $n_{c}$, and mutants, $n_{m}$, can be calculated as:

(24) $$n_{c}=2\times \lceil \frac{p_{c}\times n_{pop}}{2}\rceil$$

(25) $$n_{m}=\lceil p_{m}\times n_{pop}\rceil$$

The termination criteria for GA are the same as PSO, and NFE is defined as

(26) $$NFE\left(t\right)=n_{pop}+(n_{c}+n_{m})\times n_{it}.$$

Table 4 lists the simulation parameters for GA in different scenarios, and Algorithm 2 presents the pseudo-code of GA.

**Algorithm 2.** Genetic Algorithm.1:**Input:**$n_{pop}$: population size; $n_{var}$: no. of variables; $n_{it}$: maximum no. of iterations; $p_{c}$: crossover percentage; $p_{m}$: mutation percentage; ξ: mutation rate; η: selection pressure.2:**Output:** Best solution3:**for**$i\;=\;1:n_{pop}$**do** (initialize the parameters)4:  Randomly generate positions $X^{i}$ of $i^{th}$ individual5:  Calculate the cost value
$c_{i}$ of $i^{th}$ individual6:  Set the best solution $P_{best}$ of $i^{th}$ individual7:
**end for**
8:Sort populations based on their cost values9:Set best solution, $G_{best}$10:Set the worst cost, $c_{max}$11:Calculate the number of offsprings, $n_{c}$, using Equation (24)12:Calculate the number of mutants, $n_{m}$, using Equation (25)13:**for** i = 1: $n_{it}$ do14:  Calculate selection probability using Equation (23)15:  **for**
$k\;=\;1:n_{c}/2$ do16:    Select offsprings using RWS17:    Apply crossover18:    Calculate the cost values $c_{k}$ of offsprings19:  **end for**20:  **for**
$k\;=\;1:n_{m}$ do21:    Randomly select offspring22:    Apply mutation23:    Calculate the cost values $c_{k}$ of mutant24:  **end for**25  Create merged population26:  Sort populations based on their cost values27:  Update the worst cost, $c_{max}$28:  Truncate the population and select the first $n_{pop}$ individuals29:  Check the feasibility of the solution by checking Equations (9)–(11) = 030:  Update the best solution ever found31:
**end for**


#### 3.3.3. PSO-GA

Although GA has distinct capabilities, such as the cooperative use of different genetic operators (selection, crossover, and mutation), it still suffers from some inherent shortcomings, such as premature convergence, the poor capability of local search, and slow convergence speed. One of the main reasons for such shortcomings is the lack of information flow and collaboration between individuals. The lack of information flow makes GA an inefficient algorithm for solving many optimization problems.

Meanwhile, the PSO algorithm was developed based on the collaboration between particles to exploit the swarm intelligence. Therefore, to overcome the aforementioned issues, both GA and PSO algorithms can be combined to complement each other, in which the hybrid algorithm could improve the path planning algorithm performance. In addition, the PSO-GA algorithm would more effectively make a tradeoff between exploration and exploitation rate, and enhance the capacity of local search and global search, which consequently minimizes the probability of trapping in local optima and increases the probability of generating stable solutions. Algorithm 3 presents the pseudo-code of the PSO-GA algorithm, and Table 5 lists the simulation parameters for PSO-GA in different scenarios.

**Algorithm 3.** Hybrid PSO-GA algorithm.1:**Input:**$n_{pop}$: Population size; $n_{var}$: no. of variables; $n_{it}$: maximum no. of iterations; $n_{ga}$: maximum no. of subiterations for GA; $n_{pso}$: maximum no. of subiterations for PSO; w: inertia weight; $c_{1},\;c_{2}$: acceleration coefficients; $p_{c}$: crossover percentage; $p_{m}$: mutation percentage; ξ: mutation rate; η: selection pressure2:**Output:** Best solution3:**for***i* = 1: $n_{pop}$ do (initialize the parameters)4:  Randomly generate *n* initial positions $P_{i}\left(i=1,2,\ldots ,n\right)$ of $n_{pop}$ individuals5:  Set *n* initial velocities
$V^{i}\left(i=1,2,\ldots ,n\right)$ of $n_{pop}$ individuals to 06:  Calculate the cost value $c_{i}$ of each individual7:  Set best position, best cost, and best solution of $i^{th}$ particle8:  Update global best solution, $g_{best}$9:
**end for**
10:Sort populations based on their cost values11:Set the worst cost $c_{max}$12:Calculate the number of offsprings, $n_{c}$, using Equation (24)13:Calculate the number of mutants, $n_{m}$, using Equation (25)14:**for**$i\;=\;1:n_{it}$ do15:  **for**
$k\;=\;1:n_{pso}$ do16:    PSO algorithm (lines 10–22 of Algorithm 1)17:  **end for**18:  **for**
$k\;=\;1:n_{ga}$ do19:    GA algorithm (lines 14–28 of Algorithm 2)20:  **end for**21:  Check the feasibility of the solution by checking Equations (9)–(11) = 022:  Update the best solution ever found23:
**end for**


### 3.4. Performance Evaluation and Validation

Due to the principal design of the considered algorithms, the algorithms start by generating random solutions and then proceed with the optimization. Moreover, due to the multimodality aspect of the path planning problem, the outputs of the algorithms will not always be the same. Hence, we ran the simulations for each scenario 50 times (independently) and then analyzed the results statistically. The effectiveness of the solutions under different algorithms and scenarios was evaluated in terms of the median value, standard deviation (denoting the stability of the algorithms), and convergence speed of the algorithms.

To further compare the simulation results of different scenarios and algorithms, some other metrics, such as the ratio of path reliability, $R_{reliability}$; the ratio of infeasible solutions, $R_{infeasible}$; and execution time were considered, which can be calculated as

(27) $$R_{reliability}=\frac{L_{reliable}}{L_{total}},$$

where $L_{total}$ and $L_{reliable}$ can be calculated based on Equations (4) and (5), respectively,

(28) $$R_{infeasible}=\frac{n_{infeasible}}{n_{it}},$$

where $n_{infeasible}$ is the number of infeasible solutions generated during the optimization procedure. Infeasible solution refers to the solutions that the penalty terms in Equations (9)–(11) are not zero.

Although the execution time of algorithms depends on the hardware and software configuration of the utilized computer, and it is not the same for other devices, as a metric to compare the run time of algorithms, the considered scenarios and algorithms also were compared in terms of execution time. All the simulations in this study were implemented in MATLAB R2022a, and the simulation test environment is; OS: Windows 10 64-bit; Processor: Intel^®^ Core i5-4430; central frequency: 3 GHz; RAM: 32 GB.

To assess the effectiveness of the algorithms, the problem was simulated under three scenarios presented in Table 2. Finally, to validate the performance of the proposed optimization method, the simulation results of Scenario I were compared with the conducted aerial drive test on the 4G network at the UKM campus.

## 4. Results and Discussions

This section first discusses the results of a reliable aerial cellular communication zone based on the 4G dataset. Next, the UAV path planning optimization simulation results are presented and discussed. Finally, to validate the ability of the proposed strategy to solve 3D UAV path planning problems, the simulation results of Scenario I are compared with the results of the conducted aerial drive test.

### 4.1. Reliable Aerial Cellular Coverage

The dataset contains 8457 samples of the data of four BSs on the UKM campus, in which RSRP and RSRQ values were measured along different flight paths and heights. In the following, to demonstrate the status of a signal based on its RSRP and RSRQ level, we refer to the classifications presented in Table 6.

**Figure 5:**
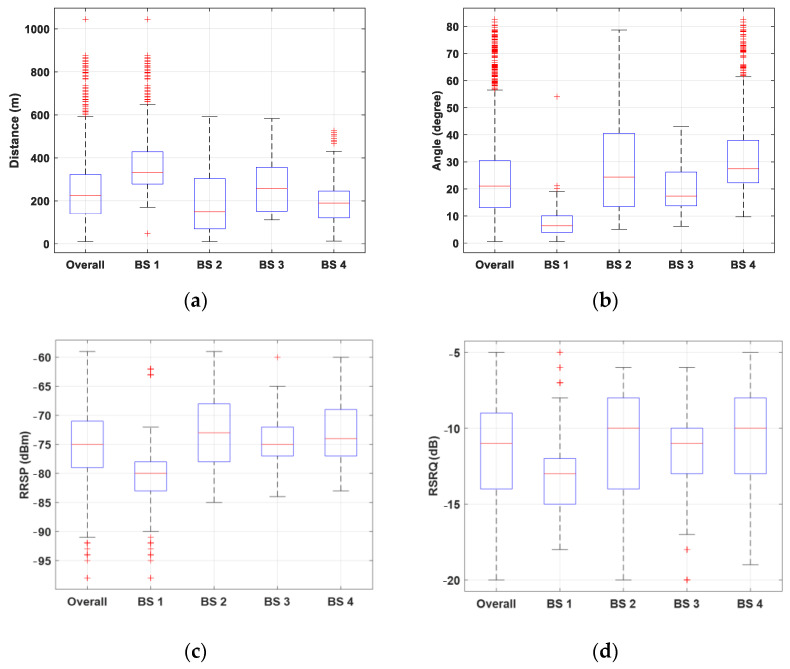
Box plot of (**a**) distance, (**b**) elevation angle, (**c**) RSRP, and (**d**) RSRQ of the overall dataset and considered BSs.

Figure 5 depicts the box plots of distance, angle, RSRP, and RSRQ for overall data as well as four considered BSs (the overall data are a combination of data from all the considered BSs). The results show that the samples are primarily located in a radius of 10 to 593 m and an angle of 0.5 to 56 degrees. The distribution of the measured data is predominantly influenced by parameters, such as network density, network design, the radiation pattern of antennas, and handover. Results in Figure 5c,d show that the received signals are in a wide range of strength and quality, from “excellent” to “weak”.

A closer look into the results of different cells reveals that, within the considered radius and elevation angle, the RSRP values between the first and third quartile are almost in “good” status. Meanwhile, RSRQ shows more variations in the considered scenarios and varies from the “good” to the “medium” range.

**Figure 6:**
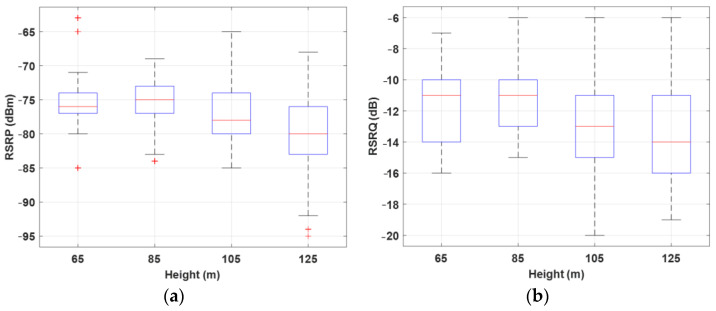
Box plot of (**a**) RSRP and (**b**) RSRQ for Route I at different heights, 65, 85, 105, and 125 m.

For a more detailed analysis, Figure 6 shows box plots of RSRP and RSRQ for Route I at different flight heights. The results show that increasing flight height decreases the median values of RSRP and RSRQ. In the conducted measurement, the drone was flying mostly in the LOS of BSs. Hence, the main factors of RSRP degradation are path loss and antenna gain reduction. The main reason for the antenna gain reduction is that the BS antennas are down-tilted; thus, based on the antenna radiation pattern, by increasing the flight height, the chance of serving the drone with the main lobe decreases. This consequently degrades the received signal energy at the drone side and enhances the uncertainty in the communication link.

On the other hand, RSRQ indicates the received signal quality in a network as well as the effect of interference from adjacent BSs. As the results in Figure 6b show, in higher altitudes, the drone considerably experiences lower link quality. The main reason is that, besides the desired signal power level degradation, in higher altitudes, drones can see a larger number of BSs. Therefore, the probability of receiving signals from adjacent/interfering BSs increases, and consequently, the drone receives higher interference energy at higher altitudes.

Another parameter that impacts the reliability of a cellular communication link is handover. Handover is considered a required procedure in cellular communications to maintain the connectivity and quality of a link between users and the network. However, the multiplicity of handovers in a short time interval shows the uncertainty of the communication link in an area. Table 7 demonstrates the impact of flight height on the number of handovers. Note that there was no continuous coverage for Route I at a height of 125 m, that is, the BSs could not provide aerial coverage for the entire path at that height. Therefore, the number of handovers was reduced. As can be seen, the number of handovers in the three routes increased significantly as the flight height increased.

**Figure 7:**
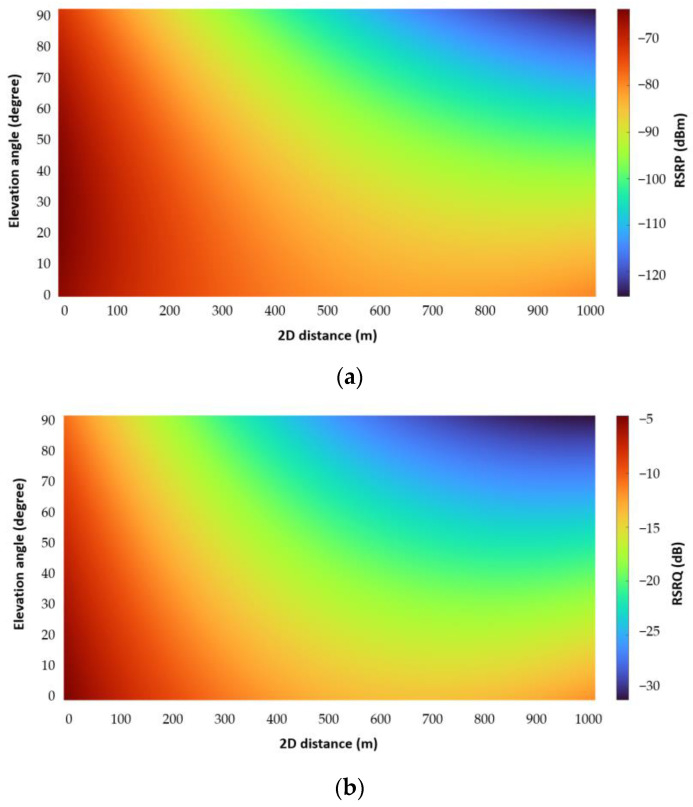
2D representation of (**a**) RSRP and (**b**) RSRQ around a 4G BS.

Figure 7 illustrates a two-dimensional representation of RSRP and RSRQ around a 4G BS, based on the RSRP and RSRQ prediction model in [51], Equations (14) and (15). Note that the utilized equations were developed based on the same dataset used in this study. The results show how RSRP and RSRQ degrade by increasing distance and flight height. In addition, based on the results and radiation pattern of directional antennas, in a radius of roughly less than 50 m, the main lobe is not able to provide sufficient coverage, especially at altitudes above the antenna height.

In conclusion, the results show that for a flight height up to 85 m above ground level (AGL) and a 2D distance up to 500 m, the considered parameters, RSRP, RSRQ, and handover, are primarily in a reliable range, where a BS can provide RSRP and RSRQ better than −80 dBm and −14 dB, respectively. Therefore, it is expected that the existing 4G network can provide a good link for telemetry communications for drones within a 2D distance between 50 to 500 m and heights up to 85 m AGL in suburban areas, where the heights of buildings, trees, and other obstacles are almost lower than the heights of BSs.

### 4.2. Path Planning Optimization

The positions of the starting point, destination, BSs, obstacles, and no-fly zones were utilized as the inputs, and PSO, GA, and PSO-GA algorithms were employed to solve the problem. Note that the presented figures in this subsection are examples of simulation results selected based on the corresponding mean values, as described earlier in the methodology, Section 3.4. A detail of the statistical results of the simulations is presented in Appendix A Table A1. In addition, to better visualize the results, the path planning results are depicted in a 2D form, a horizontal projection of the 3D path planning on xy plane, and only an example of 3D simulation results of UAV path planning is presented for Scenario III.

**Figure 8:**
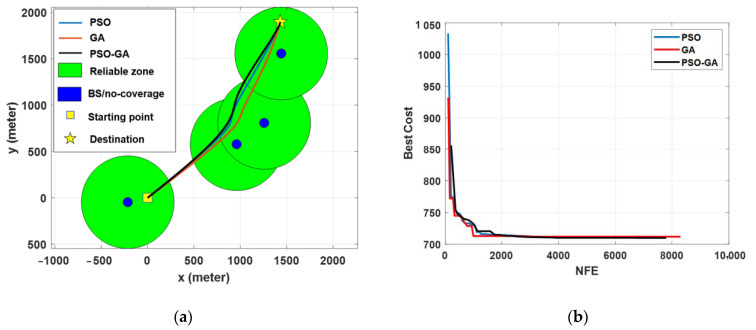
Simulation results of different optimization algorithms in Scenario I: (**a**) path planning results and (**b**) cost function value vs. the number of function evaluations.

Figure 8 presents the path planning optimization simulation results for Scenario I under different algorithms. Obviously, for an obstacle-free path, the shortest path between the starting point and destination is the direct path. However, as seen from the figure, the optimal paths pass through the reliable zones, while making a tradeoff between minimizing the traveling distance and maximizing the link reliability. By comparing the results in Figure 8a, it can be seen that PSO-GA provides a slightly better solution, in which the path is shorter while passing from the reliable zone. Results in Figure 8b show a comparison of the convergence rate of different algorithms. In a small-scale environment, the convergence speeds of all three algorithms are almost the same.

**Figure 9:**
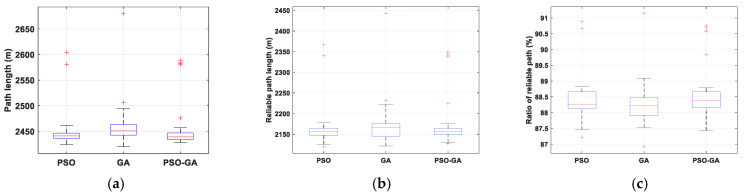
Statistical results of Scenario I: (**a**) optimal path, (**b**) reliable path, and (**c**) ratio of the reliable path.

Figure 9 shows the statistical results of path length, reliable path length, and reliable path ratio of the considered algorithms for Scenario I. The wider the data distribution (both interquartile range and whiskers), the lower the stability of the algorithms to solve the problem. In addition, a comparison between median values shows that in small-scale environments, PSO and PSO-GA algorithms slightly outperform GA in providing effective solutions for the considered problem. However, due to the simplicity of the problem in Scenario I and the distinctive features of the utilized algorithm, it can be concluded that all considered algorithms offer effective solutions for this problem.

**Figure 10:**
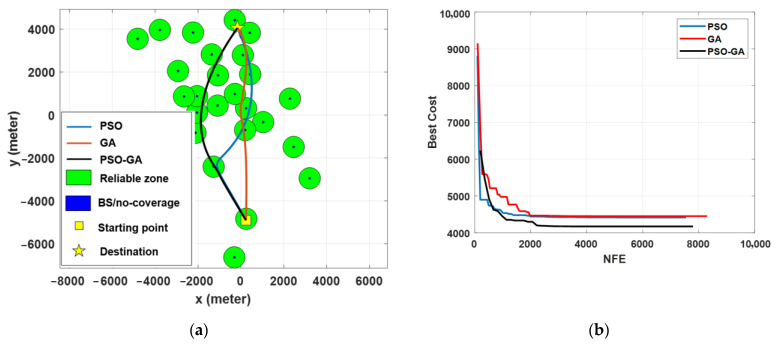
Simulation results of different optimization algorithms in Scenario II: (**a**) path planning results and (**b**) cost function value vs. the number of function evaluations.

Figure 10 presents the result of the path planning optimization in Scenario II, which corresponds to a long-range BVLOS scenario. The results show better path planning results of the PSO-GA algorithm compared with PSO and GA due to the higher exploration and exploitation capability of the PSO-GA algorithm for searching the entire search space and, consequently, do not trap in the local optimal and better converge to the global optimal. Results also show some of the shortcomings of the PSO and GA algorithms to minimize the cost function and get trapped in local minima. For example, although the designed path by GA provides a shorter path compared with the other two algorithms, for about 4 kilometers, it passes from an area without cellular coverage. In contrast, PSO-GA effectively computes a path with the most reliable coverage while also minimizing the traveling path. The results of Figure 10b show that PSO-GA also surpasses PSO and GA algorithms in terms of convergence speed.

**Figure 11:**
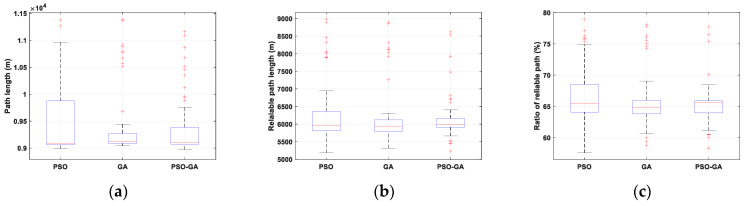
Statistical results of Scenario II: (**a**) optimal path, (**b**) reliable path, and (**c**) ratio of the reliable path.

Figure 11 compares the statistical results of the path length, reliable path length, and reliable path ratio of 50 simulation experiments on three algorithms in Scenario II. It should be noted that by increasing the environmental scale and enhancing the number of objects, such as BSs and obstacles, the multimodality of the problem will increase, in which the complexity of finding a global optimal for the problem will be increased as well.

Based on the interquartile range of presented box plots, the instability of PSO in optimizing a large-scale path planning problem is obvious. The generated paths vary from straight lines to curvy paths, such as the designed paths by GA and PSO-GA in Figure 10a, respectively. One of the reasons for this behavior is the feature of information flow in the PSO algorithm, which helps the algorithm to explore the search space better. Meanwhile, during the optimization procedure, due to the inertia, cognitive, and social acceleration coefficient adjustments, the exploration rate gradually decreases, and the exploitation rate gradually increases. This sometimes results in premature convergence, and the algorithm gets trapped in local minima.

On the other hand, although GA provides concentrated results, which look stable, it should be noted that in most simulation experiments, the algorithms converge to a local minimum and is not capable of effectively making a tradeoff between objectives, minimizing the traveling distance and maximizing the link reliability. Among all algorithms, PSO-GA provides stable solutions with a slightly better reliable path length and reliable path ratio.

**Figure 12:**
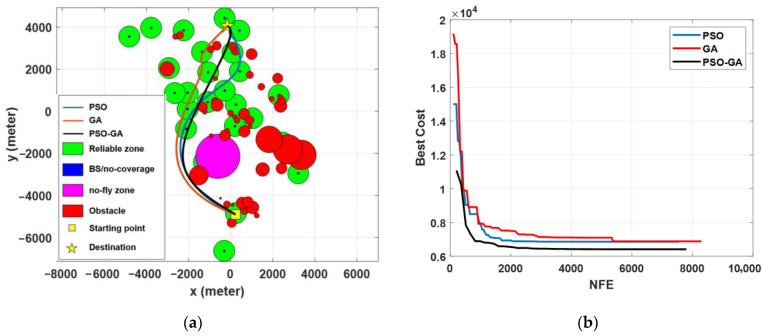
Simulation results of different optimization algorithms in Scenario III: (**a**) path planning results and (**b**) cost function value vs. the number of function evaluations.

**Figure 13:**
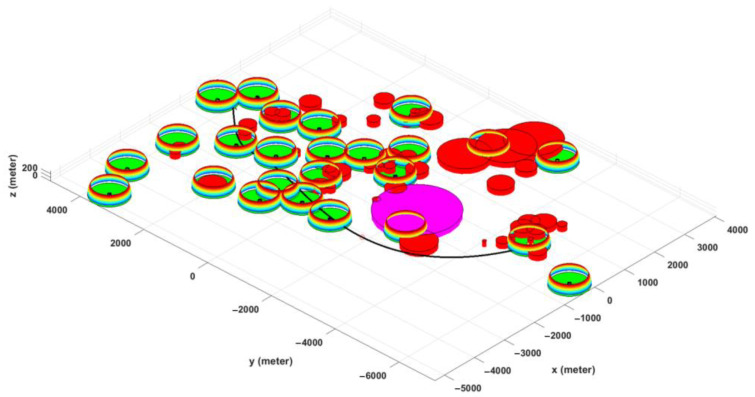
An example of a 3D representation of UAV path planning.

**Figure 14:**
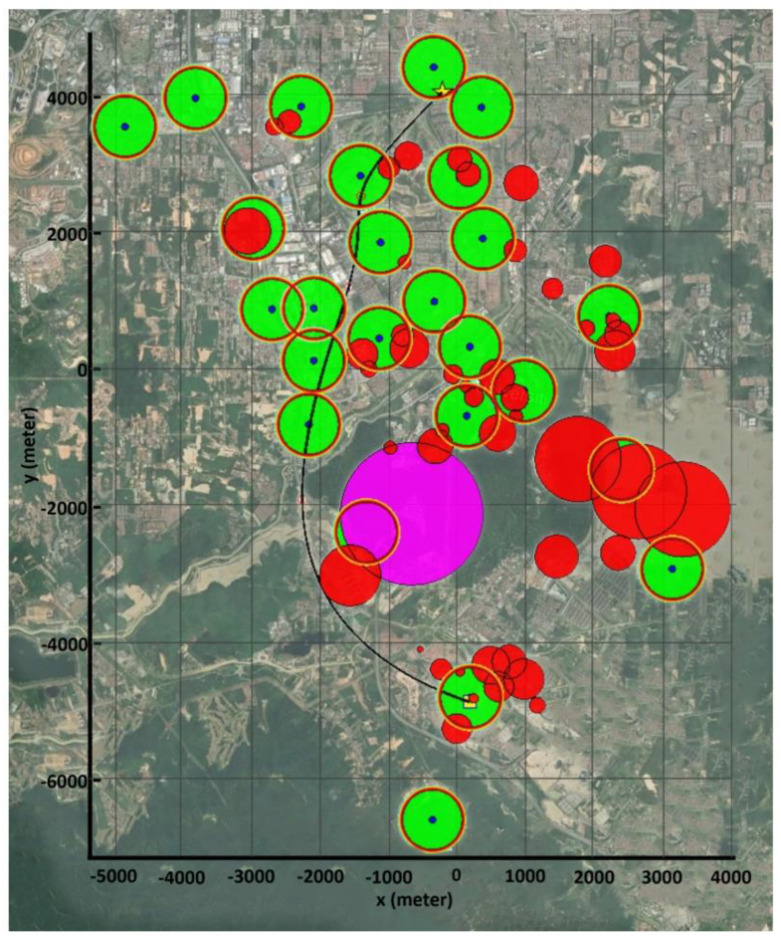
Overlaying a horizontal projection of the 3D path planning on the Google map of the considered environment.

Scenario III was developed based on a set of real-world constraints, such as the locations of BSs (in this case, the cellular network design in suburban environments), obstacles, and a no-fly zone. The added constraints considerably enhanced the complexity of the problem as well as its multimodality. Simulation results in Figure 12 show that all three algorithms are capable of computing acceptable paths for the considered problem. However, with the same justification as Scenario II, the PSO-GA algorithm still surpasses the other two algorithms in terms of generating a smoother path and faster convergence speed. Figure 13 depicts a 3D map of the UAV path planning with the PSO-GA algorithm for Scenario III, and Figure 14 depicts an overlay of the horizontal projection of the 3D path planning on the Google map of the considered environment. As can be seen from the figures, PSO finds a valid path while effectively avoiding obstacles and the no-fly zone and maximizing the reliable path length.

**Figure 15:**
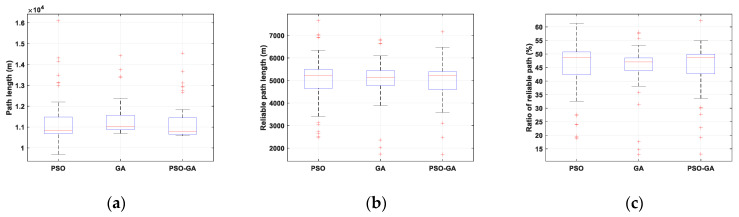
Statistical results of Scenario III: (**a**) optimal path, (**b**) reliable path, and (**c**) ratio of the reliable path.

Figure 15 compares the statistical results of the path length, reliable path length, and reliable path ratio of the three algorithms in Scenario III. By comparing the results of Figure 15a, it can be seen that the median values of all three algorithms are almost the same. However, PSO-GA surpasses the other two algorithms due to the shorter uppers and lower whiskers, as well as the closeness of the median value to the minimum computed path length. The results comparison also reveals the lower stability of PSO compared with GA and PSO-GA. By comparing the results of Scenarios II and III, it can be seen that in Scenario III, the performance of the algorithms is more similar compared with Scenario II. That is because by adding more constraints to the problem, the search space becomes more restricted, which limits algorithms’ instability in generating more diverse solutions.

**Figure 16:**
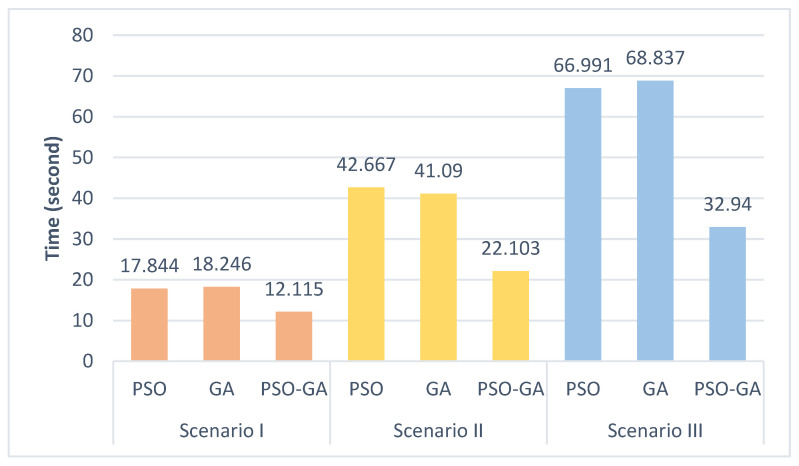
Comparison of the algorithms’ execution time in different scenarios.

As previously mentioned, enlarging the scale of the problem and increasing the number of objects in a problem will increase the complexity of the problem. Figure 16 presents the results of the average execution time of three algorithms in different scenarios. As can be seen, the execution time of PSO and GA algorithms is almost the same, while PSO-GA executes far faster than the other two algorithms, about two times in complex scenarios. This superior performance becomes even more critical when the algorithms run on low-power computing devices, such as onboard processors or embedded systems.

### 4.3. Results Validation

**Figure 17:**
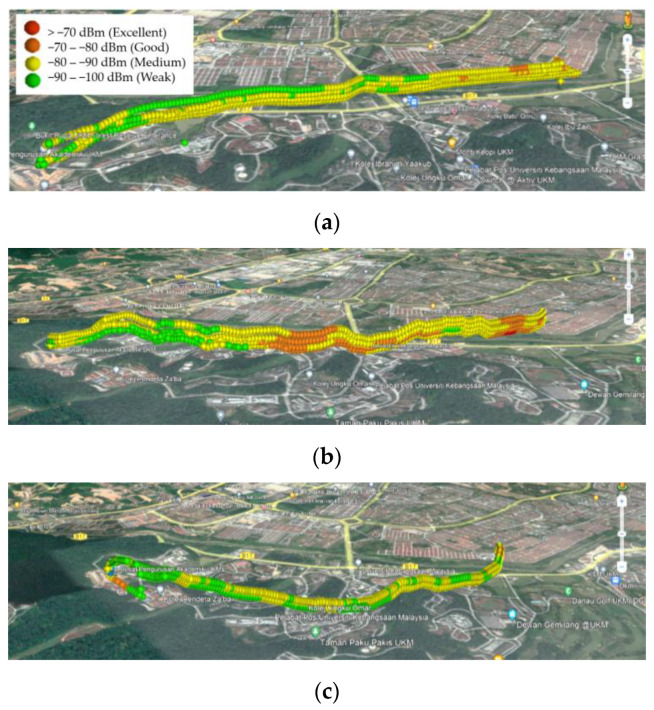
RSRP measurements for the aerial drive test on the UKM campus at different heights (65, 85, 105, and 125 m) and different routes: (**a**) Route I, (**b**) Route II, and (**c**) Route III.

**Figure 18:**
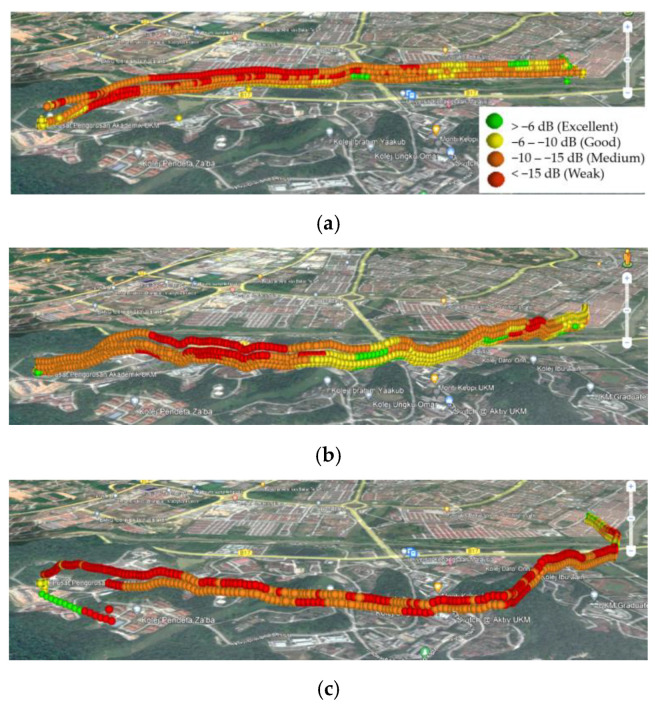
RSRQ measurements for the aerial drive test on the UKM campus at different heights (65, 85, 105, and 125 m) and different routes: (**a**) Route I, (**b**) Route II, and (**c**) Route III.

To validate the ability of the proposed strategy to solve the 3D UAV path planning problems, this subsection compares the simulation results of Scenario I with the results of the conducted aerial drive test on the UKM campus. Figure 17 and Figure 18 indicate the RSRP and RSRQ values for different routes and heights. The change of the color spectrum from cold to warm indicates the improvement of the RSRP and the weakening of the RSRQ. The result comparison reveals a better aerial coverage on Route II in terms of RSRP and RSRQ. Additionally, by comparing the results of Route II with the BSs’ locations in Figure 3, it can be revealed that the UAV is served with better RSRP and RSRQ values when it is inside the proposed reliable zone.

**Figure 19:**
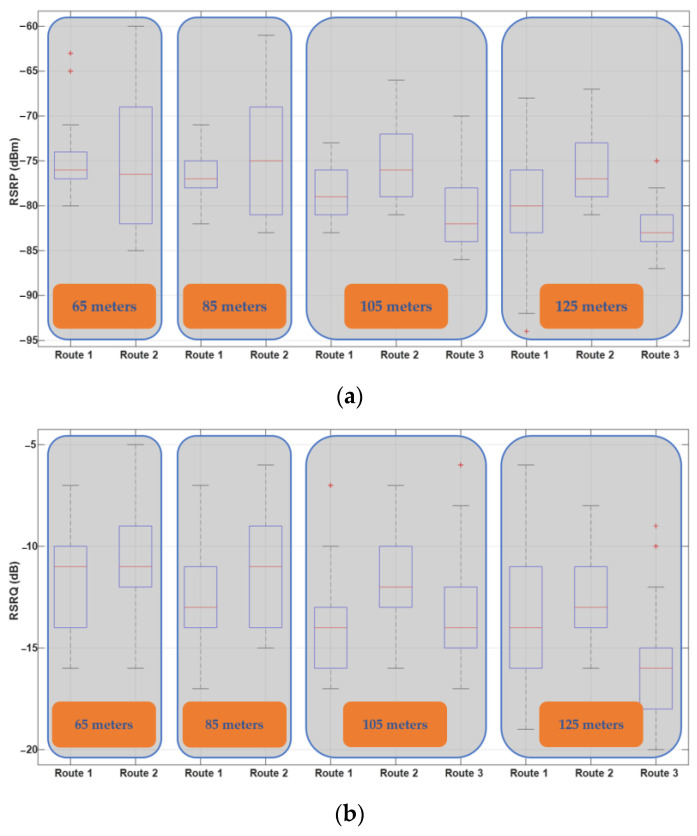
Statistical results of the conducted aerial drive test on the UKM campus at different routes and flight heights: (**a**) RSRP and (**b**) RSRQ.

Figure 19 summarizes the RSRP and RSRQ measurement results for different routes and heights in the form of box plots. As can be seen, by increasing the flight height, the mean values of both RSRP and RSRQ degrade. On the other hand, Route III provides the worst result, since throughout the flight mission, the distance between the UAV and BSs is greater compared with Routes I and II. Based on the minimum, maximum, and mean values of presented results as well as their distribution, Route II with flight heights of 65 and 85 m provides the best RSRP and RSRQ results.

**Figure 20:**
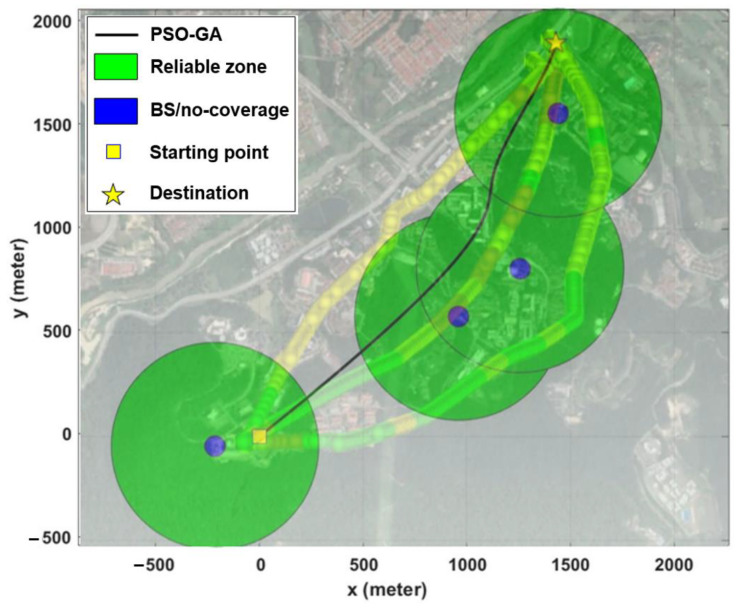
Comparison of path planning optimization simulation results against aerial drive test measurements.

Finally, Figure 20 projects the results of the optimized flight path (computed by the PSO-GA algorithm) on the results of the three measured routes (at a height of 85 m). The results reveal that the computed optimal path is close to Route II, and the proposed strategy can effectively solve the UAV path planning problem in real-world scenarios.

Generally, the results show that the PSO-GA algorithm can provide the best solution for the 3D UAV path planning problem among all three algorithms. PSO-GA has the best performance in terms of generating feasible solutions, reliable path length, reliable path ratio, convergence speed, and execution time. In addition, PSO-GA outperforms the other two algorithms in terms of searching ability and stability. The comparison of simulation and measurement results also validates the effectiveness and feasibility of the proposed solution in a suburban environment.

## 5. Conclusions

In this work, we have proposed a 3D global path planning optimization method for cellular-connected UAVs to enhance the flight safety of low-altitude UAVs. In this regard, the preexisting knowledge of a mission and environment, such as locations of starting point, destination point, BSs, obstacles, and no-fly zones, can be given as the inputs to the algorithm. The output of the algorithms is a global optimized path in the form of 3D waypoints, which can be fed to the flight controller before its mission.

The results of the conducted aerial drive test have been statistically analyzed to consider realistic constraints of cellular networks in the path planning problem. It has been revealed that within a radius of 500 m and a height of up to 85 m AGL, the existing 4G network can provide reliable aerial coverage for CC communications. The other real-world constraints, such as maximum flight distance and permitted flight height, have also been considered in the algorithm, which can be set based on the type of the UAV and the regulations of each country/region.

The PSO, GA, and PSO-GA algorithms have been used to solve the defined problem. To assess the effectiveness of the algorithms, the performance of the algorithms has been investigated under different scenarios. The simulation results revealed some shortcomings of the GA and PSO algorithms, such as getting trapped in local minima, slow convergence speed, and instability. Thus, a combination of both algorithms has been used to address the issues. The PSO-GA algorithm is empowered by the powerful aspects of two algorithms, the information flow of PSO, and the crossover and mutation operations of GA. The simulation results showed that among all the three algorithms, PSO-GA provides the best solution for the 3D UAV path planning problem, surpassing the other two algorithms in terms of stability, reliable path length, reliable path ratio, convergence speed, and execution time. Finally, the comparison between the simulation results and the measurement results proved the effectiveness and feasibility of the proposed method in solving 3D UAV global path planning in suburban environments.

The future work is to improve the accuracy of the RSRP and RSRQ models by taking into account the effect of the azimuth angle on the link reliability. Furthermore, the aerial coverage of 5G networks needs to be investigated in different scenarios, and the proposed method can be updated accordingly. Besides, the performance of the proposed model can be investigated with more state-of-the-art intelligent algorithms.

## Figures and Tables

**Table 1:** A summary of the reviewed UAV path planning models.

Ref.	Application	Environment/Scenario	Objective(s)	Approach	Key Findings	Limitations
[32]	Combat UAV path planning	Flat combat field with threats and other constraints	Minimize traveling path	ABC algorithm improved by a balance-evolution strategy	Improved performance compared with ABC by enhancing the local and global exploitation	Limited to 2D path planning and few constraints
[33]	UAV path planning during emergency landing	Different scenarios with different levels of difficulty include no-fly zone and static objects	Minimize damages and enhance safety during landing	Greedy heuristic, genetic algorithm (GA), and multipopulation GA	Genetic algorithms return better quality solutions within a reasonable computational time	The focus of the problem was on the landing procedure, not the entire path
[34]	3D UAV path planning	Digital map with few mountains or threats	Minimize the traveling path, under fuel, threat, and altitude cost functions	Improved chaos particle swarm optimization (PSO)	Overcome the inadequacy of the PSO algorithm, which falls into local optimum and slow converge	Restricted to only considering mountains
[36]	3D UAV routing with collision avoidance	3D operating spaces containing hazard areas and other moving objects	Collision avoidance	Graph theory-based algorithm	Reduced algorithm complexity and developed a novel, adaptive, graph search scheme	Parameters such as computational time and energy efficiency were not considered
[42]	General 2D UAV path planning	Static environment	Minimize energy consumption	GA with improved crossover operation	Improved convergence speed and improved GA performance	2D path planning is limited to a few constraints for terrestrial robots
[43]	3D quasi-optimal UAV path planning	Path planning for rotor wing UAVs in the complex 3D spaces	Minimize multiple cost functions: path length, std of the height, and path smoothness	Modified wolf pack search algorithm	Improved the performance of WPS by applying crossover and mutation operations of GA	Slow convergence rate, which can be due to the exploration rate, is also limited to a few constraints
[44]	UAV BVLOS path planning	Flying while accommodating real-time traffic andgeofencing constraints	Obstacle detection and avoidance	Rapidly exploring random tree planning	Suited for limited onboard processing capacity since it has simple instructions to early stop tree expansion	Specifically designed for obstacle avoidance and computationally expensive for large-scale multiobjective path planning optimization
[45]	Cellular-enabled UAV	Single UAV and multiple ground control stations	Minimizing flight time, while ensuring a sum constraint of the connectivity outage performance	Greedy algorithm based on graph theory	Proposed a low-complexity method to obtain an approximate optimal solution	2D path planning solution, also real-world constraints such as flight height constraints and obstacles were not considered
[46]	UAV-assisted cellular network	UAV works as a relay to provide downlink coverage	Maximizing total sum rate and minimizing flight time	Deep learning based	Improved the signal quality in the backhaul link of a cellular network	Constraints such as obstacles and power consumption have not been considered
[47]	UAV-assisted cellular network	UAV works as a relay to provide downlink coverage in emergency cases	Maximize system throughput by optimizing altitude and transmission power	PSO	Outperformed conventional fixed altitude and fixed transmit power approaches	Issues such as energy efficiency and the presence of obstacles have not been considered

**Table 2:** Simulation parameters for modeling environments.

Parameters	Scenario I	Scenario II	Scenario III
Environment	UKM	Bangi	Bangi
No. of BSs	4	25	25
Height of BSs (m)	40	40	40
2D distance between starting and end points (km)	2.38	8.97	8.97
Obstacle	No	No	Yes
No-fly zone	No	No	Yes

**Table 3:** General and PSO simulation parameters.

Parameter	Scenario I	Scenario II	Scenario III
Reliable zone radius (m)	500	500	500
No coverage zone (m)	50	50	50
$x_{min}/x_{max}$	−500/2200	−6000/6000	−6000/6000
$y_{min}/y_{max}$	−500/2200	−7000/5000	−7000/5000
$z_{min}/z_{max}$	65/85	65/85	65/85
α in Equation (3)	0.8	0.8	0.8
β, γ, δ in Equation (3)	10^5^	10^5^	10^5^
$n_{it}$	150	150	150
$n_{pop}$	50	50	50

**Table 4:** Parameters for GA simulations in different scenarios.

**Parameter**	$n_{pop}$	$n_{it}$	$p_{c}$	$p_{m}$	ξ	η
**Value**	50	150	0.8	0.3	0.02	8

**Table 5:** Simulation parameters for the PSO-GA algorithm in different scenarios.

**Parameter**	$n_{pop}$	$n_{it}$	$n_{pso}$	$n_{ga}$
**Value**	50	50	2	1

**Table 6:** Signal status based on RSRP and RSRQ values [56].

Signal Strength/Quality	RSRP	RSRQ
Excellent	−60–−70 dBm	>−6 dB
Good	−70–−80 dBm	−6–−10 dB
Medium	−80–−90 dBm	−10–−15 dB
Weak	−90–−100 dBm	<−15 dB

**Table 7:** The number of handovers in different routes and flight heights.

Height (m)	Route I	Route II	Route III
65	7	7	-
85	8	8	-
105	13	10	9
125	6	10	12

## Data Availability

The utilized dataset in this study is publicly available on: Mehran Behjati, 2022, UAVCellularDataset_suburban, GitHub, https://github.com/Mehranbjt/UAVCellularDataset_Suburban (accessed on 16 September 2022).

## Appendix A

**Table A1:** Statistical results of the simulations under different scenarios.

		Scenario I			Scenario II			Scenario III		
Parameters	Metrics	PSO	GA	PSO-GA	PSO	GA	PSO-GA	PSO	GA	PSO-GA
Optimal Path Length	Mean (km)	2.446	2.457	2.451	9.511	9.458	9.412	11.398	11.631	11.208
	Median (km)	2.441	2.451	2.439	9.092	9.121	9.110	10.848	11.020	10.783
	Min (km)	2.424	2.420	2.428	8.989	9.044	8.973	9.687	10.692	10.579
	Max (km)	2.604	2.680	2.589	11.378	11.394	11.160	16.096	14.424	14.548
	Std (m)	31.198	35.874	40.497	721	692	608	1219	830	918
	Skewness	4.280	4.929	2.872	1.280	1.722	1.639	1.844	2.101	1.919
	Kurtosis	20.8757	31.004	9.706	3.074	4.343	4.358	6.502	6.990	6.013
Reliable Path Length	Mean (km)	2.163	2.169	2.171	6.395	6.282	6.165	5.086	5.420	4.959
	Median (km)	2.157	2.166	2.157	5.955	5.925	5.987	5.209	5.138	5.219
	Min (km)	2.120	2.121	2.127	5.184	5.177	5.234	2.479	1.738	1.720
	Max (km)	2.367	2.443	2.349	8.984	8.898	8.618	7.649	6.821	7.160
	Std (m)	41.460	45.610	53.338	1030	939	678	1193	993	952
	Skewness	3.988	4.433	2.732	1.232	1.681	2.225	−0.352	−1.327	−1.007
	Kurtosis	19.269	27.085	9.148	3.102	4.364	8.132	3.179	6.275	5.220
Ratio of Path Reliability	Mean (%)	88.391	88.266	88.545	66.869	66.106	65.381	45.110	44.740	44.786
	Median (%)	88.270	88.227	88.380	65.386	64.767	65.562	48.582	47.067	48.787
	Min (%)	87.213	86.922	87.434	57.568	58.681	58.327	18.905	12.939	13.111
	Max (%)	90.875	91.142	90.754	78.965	78.096	77.715	61.262	58.022	62.250
	Std	0.621	0.601	0.732	5.522	4.752	3.715	10.834	8.973	9.653
	Skewness	1.970	2.032	1.900	0.806	1.262	1.306	−1.000	−2.131	−1.452
	Kurtosis	9.713	11.891	6.383	2.740	3.789	6.044	3.408	8.032	4.872
Execution Time	Mean (s)	17.844	18.246	12.115	42.667	41.090	22.103	66.991	68.837	32.940
Final Solution Feasibility	(%)	100	100	100	100	100	100	98	100	100
